# Haplotype-resolved chromosome-level genome assembly of creeping bentgrass, *Agrostis stolonifera*

**DOI:** 10.1038/s41597-026-06561-4

**Published:** 2026-01-16

**Authors:** Matthew D. Robbins, Sunchung Park, B. Shaun Bushman, Scott E. Warnke, Jinyoung Y. Barnaby

**Affiliations:** 1 United States Department of Agriculture, Agricultural Research Service, Forage and Range Research Unit, Logan, UT 84322 USA; 2 United States Department of Agriculture, Agricultural Research Service, Beltsville Agricultural Research Center, Sustainable Perennial Crops Laboratory, Beltsville, MD 20705 USA; 3 United States Department of Agriculture, Agricultural Research Service, United States National Arboretum, Floral and Nursery Plants Research Unit, Beltsville, MD 20705 USA

**Keywords:** Genome, Polyploidy in plants

## Abstract

Creeping bentgrass (*Agrostis stolonifera*) is a widely used cool-season turfgrass valued for its fine texture and ability to form dense, uniform turfs. However, its complex allotetraploid genome and high repetitive content have posed challenges for genomic research and molecular breeding. Here, we report a haplotype-resolved chromosome-level genome assembly generated using PacBio HiFi and Oxford Nanopore sequencing with Omni-C scaffolding. The final assembly spans 5.4 Gb, with a scaffold N50 of 187.9 Mb and comprises 28 pseudochromosomes representing fully phased haplotypes (2n = 4x = 28). BUSCO analysis indicated 98.8% completeness, indicating the high quality of the assembly. We annotated 146,216 protein-coding genes and found that transposable elements account for 79.8% of the genome, dominated by LTR-Gypsy elements. Subgenome-specific LTR clustering and comparative genomic alignments supported an allopolyploid origin involving two diverged progenitors. This high-quality genome provides a foundational resource for functional genomics and breeding efforts to improve disease resistance, abiotic stress tolerance, and turf quality.

## Background & Summary

*Agrostis stolonifera* L. (creeping bentgrass) is a cool-season, perennial turfgrass widely cultivated in temperate regions for high-value turf applications such as golf course putting greens, tees, and fairways^1–3^. Its fine leaf texture, tolerance to low mowing heights, and vigorous lateral spread through stolons allow it to form uniform, high-density turf under intensive management^4^. These traits have also made it a key breeding target for improving wear tolerance, aesthetic quality, and overall turf performance under stress conditions. As turfgrass systems face increasing pressure from limited water availability, high temperatures, and other environmental stressors, the ability of creeping bentgrass to maintain growth and recover from damage has become increasingly valuable for sustainable turf management^5,6^.

Creeping bentgrass is a highly outcrossing allotetraploid (2n = 4x = 28) with the genome designation A_2_A_2_A_3_A_3_^7^. The diploid progenitors that contributed to the A_2_ and A_3_ subgenomes remain unknown. The application of genomics-assisted breeding in creeping bentgrass has been constrained by the biological complexity of its genome. *A. stolonifera* exhibits high levels of heterozygosity and repetitive DNA, which have posed substantial challenges for genome assembly, gene annotation, and subgenome resolution^7^. As a result, although creeping bentgrass is among the most commercially important turfgrass species, its genetic improvement has primarily relied on traditional breeding methods, with limited capacity to apply molecular tools to identify or manipulate genes associated with key traits such as drought tolerance, disease resistance, and vegetative propagation.

Importantly, *A. stolonifera* also holds a unique place in turfgrass biotechnology history as the first turfgrass species to undergo successful genetic transformation. Early studies demonstrated stable gene insertion and expression using *Agrobacterium*-mediated and particle bombardment methods, establishing foundational protocols for turfgrass transformation and regeneration^8–10^. These efforts led to the development of transgenic lines with novel traits such as herbicide tolerance and offered proof-of-concept for applying molecular breeding techniques in perennial grass species. The technical advances from this body of work continue to inform current strategies in turfgrass genomics, gene discovery, and functional validation.

Over the past two decades, a number of foundational studies have contributed important genetic and genomic resources for *A. stolonifera*. Molecular markers—including isoenzymes, restriction fragment length polymorphism (RFLPs), amplified fragment length polymorphisms (AFLPs), random amplified polymorphic DNA (RAPDs), and simple sequence repeat (SSRs)—have revealed considerable genetic diversity across cultivars, germplasm collections, and related species^11–23^. Linkage mapping and QTL analyses have identified genomic regions associated with dollar spot resistance and summer stress performance, providing a basis for marker-assisted selection^24–27^. Cytogenetic work has clarified ploidy variation and genome structure within the genus^19,20,28–30^.

Organellar genomes have further advanced our understanding of *A. stolonifera* evolution. Complete chloroplast and mitochondrial genomes have been sequenced and compared across grass species to investigate codon usage, gene content, and genome structure^31,32^. Comparative analyses between *A. stolonifera* and other Pooideae members—such as *Brachypodium distachyon*, *Hordeum vulgare*, and *Lolium perenne*—have revealed conserved synteny and evolutionary relationships across lineages^33^. Although the synteny-based draft genome of *L. perenne* has served as a useful reference for comparative work^34^, its evolutionary distance from *Agrostis* limits its utility for trait discovery or subgenome-level analysis in creeping bentgrass.

Transcriptomic and gene expression studies have provided additional insights into creeping bentgrass responses to abiotic stresses. Drought and heat stress have been shown to induce widespread changes in gene expression, including those related to polyamine biosynthesis, cytokinin regulation, and oxidative stress response^35–37^. Other studies have explored the transcriptional basis of cadmium tolerance^38^, and machine learning–based annotation tools have been used to classify creeping bentgrass proteins and improve functional predictions^39^. Together, these efforts have significantly advanced our understanding of creeping bentgrass biology and established a strong foundation for future genetic improvement.

However, a major bottleneck remains: the absence of a haplotype-resolved, chromosome-scale nuclear genome. Existing genomic resources—though valuable—are fragmented, incomplete, or lack the resolution necessary to study subgenome-specific organization, repetitive element dynamics, and structural variation. Without a reference-quality genome, it has been difficult to implement genome-wide selection, conduct gene discovery at scale, investigate polyploid genome evolution, or fully leverage transgenic *Agrostis* lines, where a complete genome is essential for identifying gene targets, mapping insertion sites, and enabling precise trait engineering. This gap has limited the integration of modern genomic tools into creeping bentgrass breeding, despite growing interest in applying biotechnology and genomics to turfgrass improvement^40,41^.

To address these challenges, we developed a chromosome-level, haplotype-resolved genome assembly of creeping bentgrass using PacBio HiFi and Oxford Nanopore long-read sequencing with Omni-C scaffolding. This resource provides the first fully phased nuclear genome for *A. stolonifera*, resolving 28 pseudomolecules corresponding to its allotetraploid genome. The assembly supports detailed annotation of protein-coding genes and repetitive elements and enables subgenome-specific comparisons that were previously not possible. By building upon the extensive body of prior work, this genome represents a critical step forward in turfgrass genomics and offers a foundational platform for evolutionary analysis, functional gene discovery, and genomics-enabled breeding.

The objectives of this study were to (i) generate a high-contiguity, chromosome-scale reference genome of *Agrostis stolonifera*; (ii) annotate gene content and repetitive sequences with subgenome-level resolution; and (iii) provide an essential genomic resource to support trait discovery, molecular breeding, and comparative biology in cool-season polyploid turfgrasses.

## Methods

### Plant material and sequencing

The creeping bentgrass plant used for genome sequencing belonged to the cultivar ‘Declaration’ and was cultivated at the Beltsville Agricultural Research Center (BARC) in Beltsville, Maryland, USA (39°02′N, 76°53′W). The seeds were originally provided by Rutgers University. Plants were grown under controlled greenhouse conditions with day and night temperatures maintained at 24 °C and 16 °C, respectively. Fresh leaf tissue was collected from a single individual plant for DNA extraction. High molecular weight genomic DNA was isolated using a modified plant extraction method^42^ adjusted with an extended incubation in phenol followed by two chloroform extractions. DNA concentration and purity were assessed using a NanoDrop 2000 spectrophotometer (Thermo Fisher Scientific, USA). PacBio HiFi libraries were prepared and sequenced on a Sequel II instrument at the Brigham Young University (BYU) DNA Sequencing Center (Provo, UT, USA). PacBio sequencing generated 15.9 million HiFi reads, totaling 221.7 Gb, with an average read length of 13.9 kb. The high molecular weight genomic DNA extracted for PacBio sequencing was also used for Oxford Nanopore long read sequencing (ONT). Libraries were prepared with a Ligation Sequencing DNA kit and sequenced with three LSK-114 flow cells on a MinION Mk1B, yielding 480,471 reads totaling 13.3 Gb, with an average read length of 27.8 kb. A proximity ligation library was prepared using Omni-C technology (Dovetail Genomics), generating 417 million 2 × 150 bp read pairs and 124.9 Gb of sequencing data.

### Genome assembly and chromosome scaffolding

**Fig. 1:**
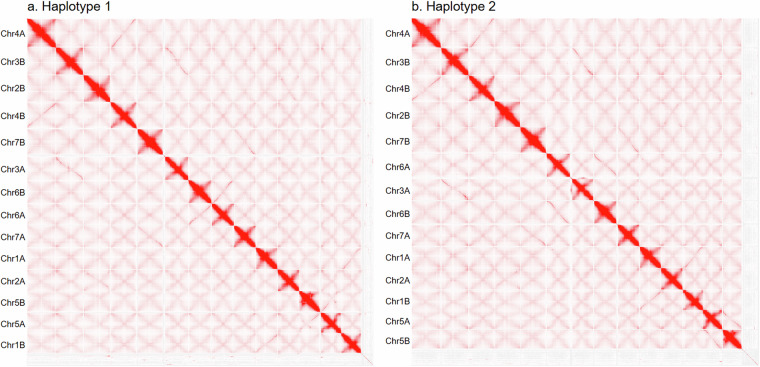
Omni-C contact maps of *Agrostis stolonifera* haplotypes. Chromosomes are shown as scaffolded within each haplotype after visual inspection with manual correction and prior to filtering, gap filling, and reorientation.

The initial assembly was generated using hifiasm v0.24.0-r702^43^ by incorporating PacBio HiFi reads, ONT long reads (–ul flag), and Omni-C paired-end reads (–h1 and–h2 flags), with a svalue (-s flag) of 0.4, resulting in fully phased haplotype groups of contigs. Contigs were scaffolded using the Omni-C reads mapped with the Arima Genomics pipeline (https://github.com/ArimaGenomics/mapping_pipeline) and assembled with YaHS v1.2.2^44^. Further refinement of the assembly involved visual inspection and manual correction of the Omni-C contact maps using Juicebox v1.11.08^45,46^ (Fig. 1), followed by reprocessing with the juicer utility in YaHS. Scaffolds were further filtered to remove contaminants, organellar sequences, and highly repetitive sequences as previously described Robbins *et al*.^42^. Gap closure was performed using TGS-GapCloser2 v1.2.1^47^ using the PacBio HiFi reads to improve continuity. Gap fills were accepted only when supported by uninterrupted coverage of PacBio HiFi and ONT reads, verified through visual inspection in Persphone v10.3.9281 (https://persephonesoft.com). Read mappings were performed with minimap2 v2.29-r1283^48^ with the *map-hifi* preset for PacBio HiFi and *map-ont* for ONT.

**Fig. 2:**
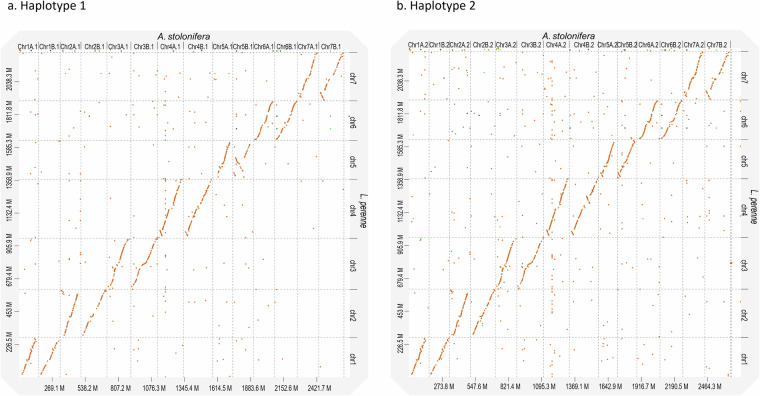
Dot plot comparison between *Agrostis stolonifera* haplotypes and *Lolium perenne* chromosomes. Pseudochromosomes from each haplotype of the tetraploid *A. stolonifera* genome assembly are shown on the x-axis, while the *L. perenne* haploid reference genome (GCF_019359855.2) is shown on the y-axis. Diagonal lines indicate regions of sequence similarity.

The final assembly consisted of 402 scaffolds, with 28 pseudochromosomes representing four haplotypes for each of seven ancestral chromosomes, totaling 5.4 Gb, with a scaffold N50 of 187.9 Mb. (Table 1). Chromosomes were oriented and numbered based on alignment to the *Lolium perenne* Kyuss v2.0 genome^49^. Alignments were generated using D-GENIES^50^ with minimap2 as the aligner and visualized using the ‘hide noise’ feature and a minimum identity threshold of 0.25 (Fig. 2).

**Table 1:** Statistics of the creeping bentgrass assemblies.

Category	Metric	Fully phased tetraploid	Subgenome A	Subgenome B
Assembly statistics	Total assembly length (Mb)	5,429	2,506	2,788
	Number of scaffolds	402	NA^§^	NA^§^
	Scaffold N50 (Mb)	187.9	NA	NA
	Pseudochromosomes (Mb)	5,294	2,506	2,788
Gene annotation	Total predicted genes	146,216	NA	NA
	Genes in Pseudochromosomes	145,556	75,862	69,694
BUSCO genome (poales_odb12)	Complete	98.8%	97.4%	97.8%
	Single-copy	0.7%	2.3%	2.8%
	Duplicated	98.0%	95.0%	95.0%
	Fragmented	0.6%	1.1%	1.0%
	Missing	0.6%	1.6%	1.1%
	Total	6,282	6,282	6,282
BUSCO annotation (poales_odb1 2)	Complete	99.5%	98.2%	98.3%
	Single-copy	0.4%	3.3%	4.8%
	Duplicated	99.1%	94.9%	93.5%
	Fragmented	0.1%	0.2%	0.3%
	Missing	0.5%	1.6%	1.4%
	Total	6,282	6,282	6,282

^§^Not applicable

### Structural variation and Subgenome differentiation

**Fig. 3:**
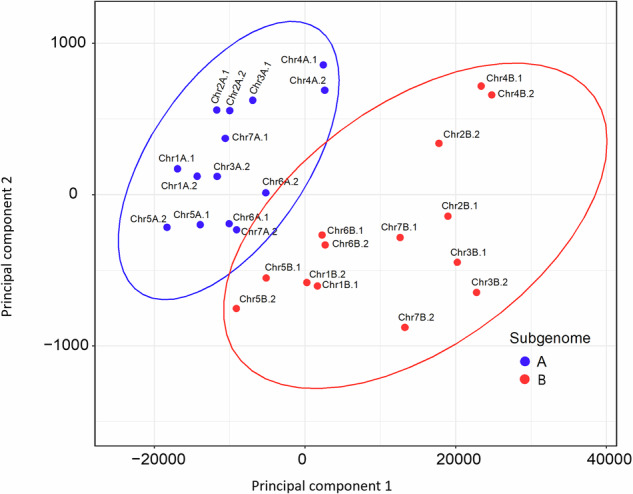
Principal component analysis (PCA) of creeping bentgrass chromosomes based on LTR Copia and Gypsy abundance and cumulative size. PCA was performed using the frequency and total occupied size of LTR-Copia and LTR-Gypsy elements per chromosome. Each point represents a chromosome, colored by its subgenome assignment (blue: A, red: B). Ellipses represent 95% confidence intervals around each subgenome cluster.

**Fig. 4:**
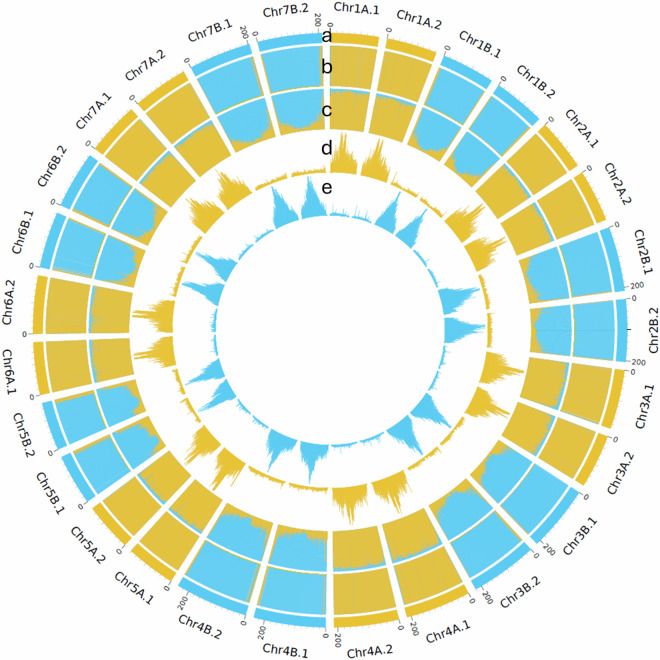
Chromosome characteristics based on differential k-mer analysis. (**a**) Chromosomes (length in Mb) with subgenome assignments based on k-means algorithm – yellow is A subgenome and blue is B subgenome. (**b**) Subgenome-specific k-mer enrichment – yellow indicates locations of significant enrichment for A subgenome-specific k-mers, blue indicates locations of B subgenome-specific k-mers. (**c**) Normalized proportion of subgenome-specific k-mers. (**d**) Absolute count of A subgenome-specific k-mers. (**e**) Absolute count of B subgenome-specific k-mers.

To assign subgenomes to the haplotypes in *A. stolonifera*, we employed a combination of FastANI^51^, LTR retrotransposon clustering, and differential k-mer analysis. FastANI was used to calculate pairwise average nucleotide identity (ANI) among pseudochromosomes. These metrics enabled clustering of scaffolds into distinct subgenome groups based on sequence similarity **(**Table 2). Subgenome assignments were further refined by analyzing the distribution of LTR retrotransposons. For each haplotype chromosome, the frequency and cumulative size of Gypsy and Copia elements were estimated and subjected to principal component analysis (PCA), which distinguished subgenome groups according to repetitive element profiles (Fig. 3; Table 3). Finally, assignments were validated by differential k-mer analysis using SubPhaser v1.2.6^52^ with default parameters. SubPhaser counts k-mers, identifies those enriched in specific subgenomes, and creates Circos plots to visualize the distribution of subgenome specific k-mers across chromosomes (Fig. 4).

**Table 2:** Pairwise Average Nucleotide Identity (ANI) among phased haplotypes of creeping bentgrass chromosomes.

Chromosome	Intra-SubgenomeA^a^	Intra-SubgenomeB^a^	Inter-Subgenome^a^
Chr1	97.68	97.83	88.42
Chr2	98.46	97.60	88.17
Chr3	97.15	98.07	88.53
Chr4	97.29	97.80	88.73
Chr5	97.87	96.98	88.33
Chr6	96.79	97.13	88.62
Chr7	98.03	97.82	88.29

^a^Intra-SubgenomeA/B refer to ANI between the two phased haplotypes of each respective subgenome. Inter-subgenome represents the average ANI between haplotypes from opposite subgenomes.

**Table 3:** Summary of chromosome-level genome assembly of creeping bentgrass.

Subgenome A		Subgenome B	
Chromosome	Length (bp)	Chromosome	Length (bp)
Chr1A.1^§^	159,129,184	Chr1B.1	174,585,626
Chr1A.2	165,773,721	Chr1B.2	170,634,216
Chr2A.1	169,190,931	Chr2B.1	211,696,985
Chr2A.2	171,329,144	Chr2B.2	204,637,557
Chr3A.1	194,775,553	Chr3B.1	215,291,366
Chr3A.2	177,043,413	Chr3B.2	220,522,120
Chr4A.1	214,301,203	Chr4B.1	235,987,658
Chr4A.2	212,667,180	Chr4B.2	239,585,240
Chr5A.1	163,072,171	Chr5B.1	167,569,048
Chr5A.2	159,093,902	Chr5B.2	158,839,814
Chr6A.1	172,882,267	Chr6B.1	185,175,001
Chr6A.2	190,797,016	Chr6B.2	187,880,091
Chr7A.1	174,094,489	Chr7B.1	204,665,110
Chr7A.2	182,026,273	Chr7B.2	211,158,997

^§^The tetraploid genome is organized into two subgenomes (A and B), each containing two phased haplotypes (denoted by the suffix ‘.1’ or ‘.2’).

**Fig. 5:**
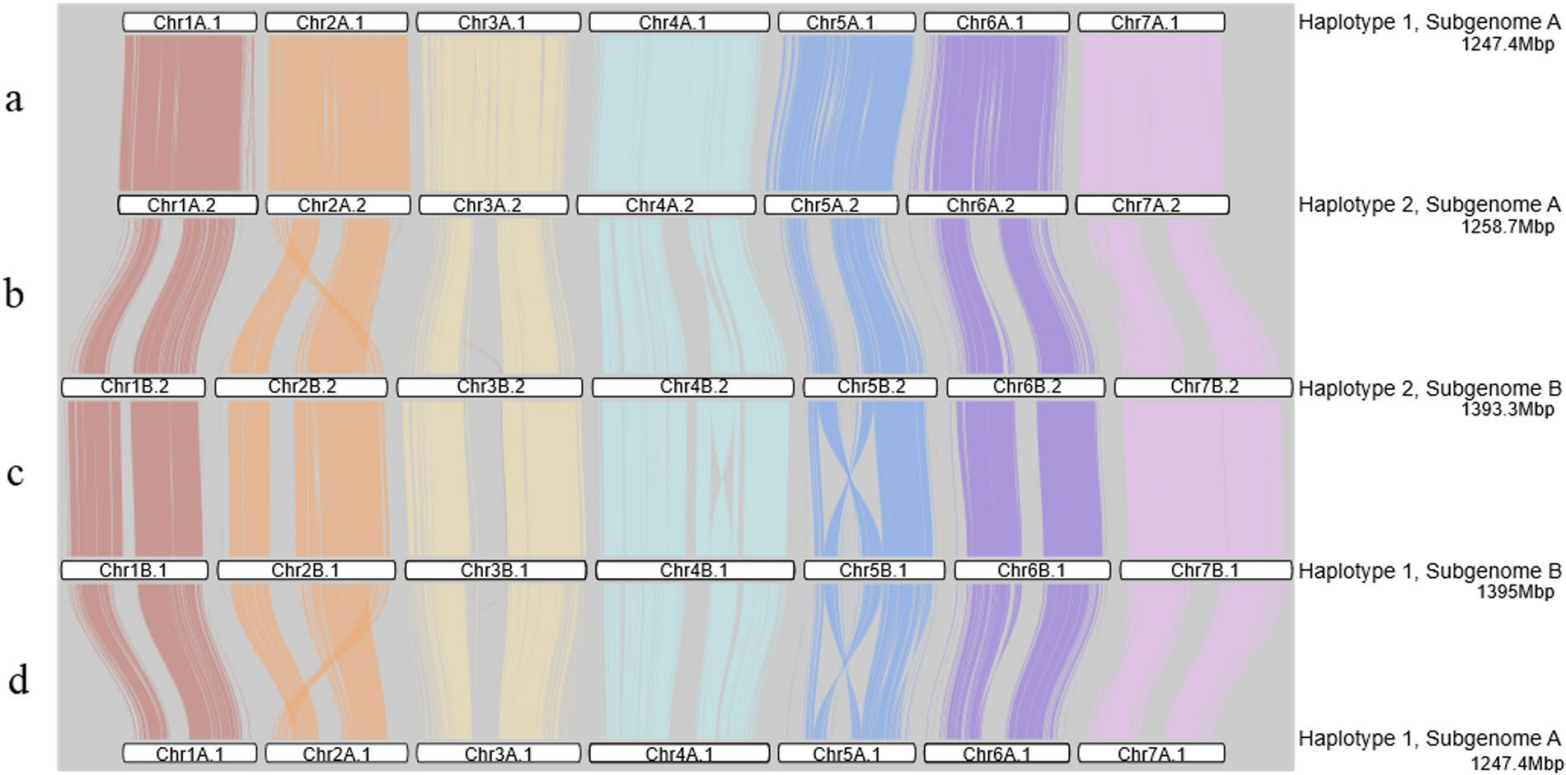
Synteny between haplotypes and subgenomes of the *A. stolonifera* genome sequence. Comparisons are arranged from top to bottom to illustrate: (**a**) synteny between haplotypes 1 and 2 of subgenome A, (**b**) between subgenome A and B in haplotype 2, (**c**) haplotypes 1 and 2 of subgenome B, and (**d**) subgenome B and A in haplotype 1.

**Fig. 6:**
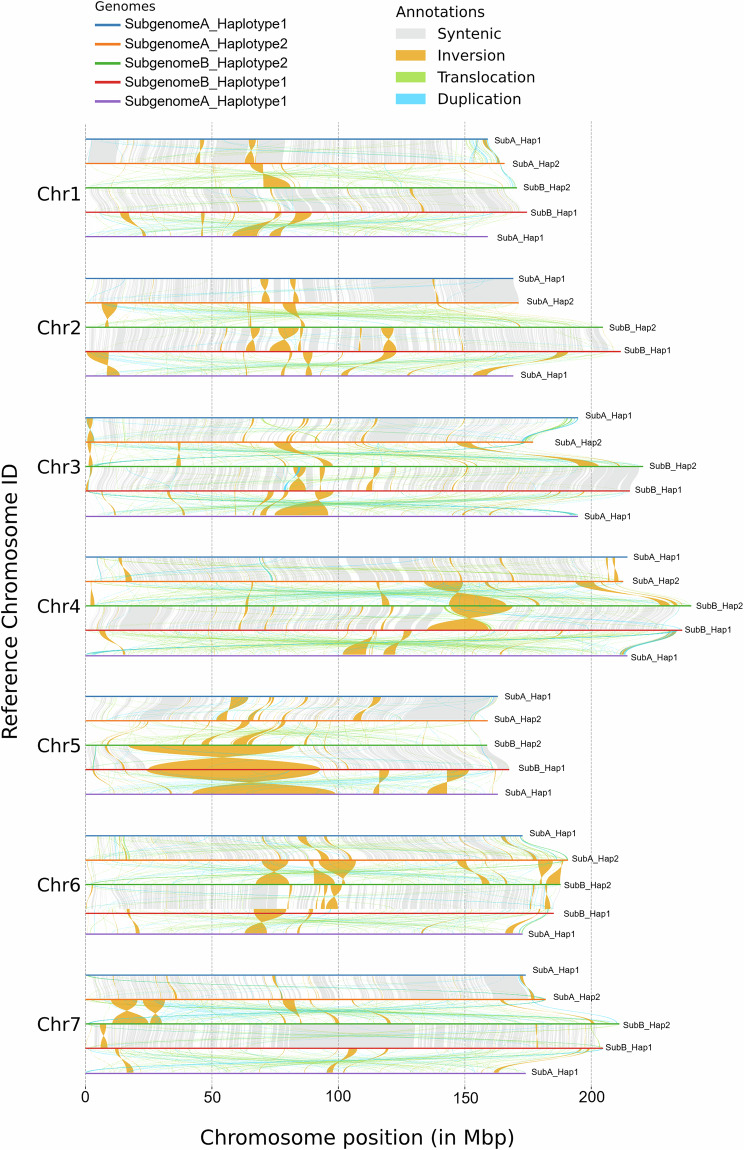
Synteny analysis of the *A. stolonifera* genome. The comparisons illustrate conserved syntenic regions across haplotypes and subgenomes. Chromosomes are displayed from top to bottom, with each panel showing: (1) synteny between haplotypes 1 and 2 of subgenome A, (2) between subgenome A and B in haplotype 2, (3) haplotypes 1 and 2 of subgenome B, and (4) subgenome B and A in haplotype 1.

The level of synteny between subgenomes and haplotypes was evaluated using DEEPSPACE v0.1 (https://github.com/jtlovell/DEEPSPACE) with the ‘close’ preset (Fig. 5). As expected, higher synteny was observed between haplotypes 1 and 2 than between subgenomes A and B. For haplotypes 1 and 2, a total of 700,659 syntenic blocks were identified across all chromosomes, with 0.890 average identity over 231 Mb. In contrast, comparisons between subgenomes A and B revealed 72,593 syntenic blocks with 0.765 average identity over 6.9 Mb. Structural variations (SV) were further characterized by aligning subgenomes and haplotypes to each other using minimap2 with the *-ax asm5* preset and*–eqx* flag, followed by SV calling with SyRI v1.7.0^53^ and visualization with plotsr v1.1.5^54^ (Fig. 6). Across all chromosomes, 349 inversions and 10,239 translocations were identified between haplotypes 1 and 2, while 2,829 inversions and 17,637 translocations were identified between subgenomes A and B, illustrating the higher similarity between haplotypes than subgenomes.

### RNA-seq and Iso-seq data

An RNA-seq dataset previously described (Amundsen *et al*. 2020)^37,55^ from cultivars ‘Declaration’ and ‘Providence’ under drought and control conditions was used as evidence for gene prediction. Reads were quality-checked and trimmed as previously described Robbins *et al*. (2025)^42^. Iso-Seq libraries were prepared from clonal plants derived from the same ‘Declaration’ individual used for whole genome sequencing, grown under standard greenhouse conditions as well as salt stress and cold stress treatments as previously described Robbins *et al*.^42^. Two 8 M SMRT cells were sequenced on a PacBio Sequel II instrument at the BYU DNA Sequencing Center (Provo, UT, USA), yielding 6 million reads totaling 11.3 Gb. Full-length transcripts were generated from HiFi reads using the PacBio Iso-Seq pipeline with default parameters.

### Gene prediction and repeat annotation

**Fig. 7:**
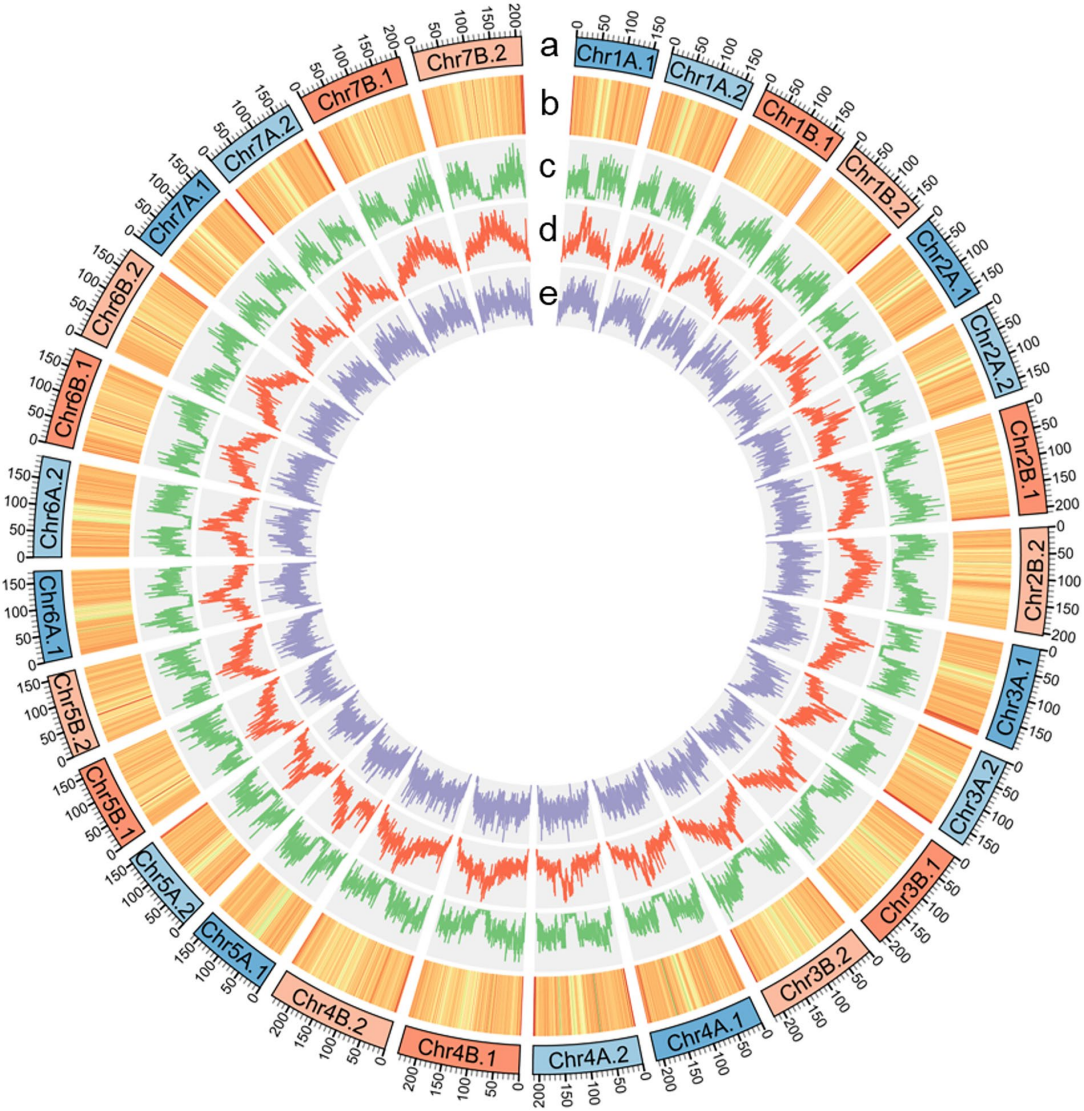
Circos plot illustrating genomic features of *Agrostis stolonifera*. (**a**) Chromosomes are arranged in a circular layout with labels and scale ticks; numbers above each chromosome represent genomic positions in megabases (Mb). (**b**) GC content is displayed as a heatmap, with a green (low)-yellow-red (high) gradient. (**c**) Gene frequency distribution. (**d**) Frequency of *Gypsy* transposable elements. (**e**) Frequency of *Copia* transposable elements. All feature values were estimated in 500 kb windows along the chromosomes.

Gene prediction was performed using an integrative approach that combined homology-based evidence, transcriptome data, and *ab initio* predictions. Homology-based annotation was conducted using GeMoMa v1.9^56^, aligning transcript sequences from four Poaceae species: *Hordeum vulgare* (GenBank Assembly Accession GCF_904849725.1), *Oryza sativa* (GCF_034140825.1), *Brachypodium distachyon* (GCF_000005505.3), and *Lolium perenne* (GCF_019359855.2). Transcriptome-based gene prediction employed a pipeline integrating STAR v2.7.11b^57^, StringTie v 2.2.0^58^, and PASA v2.5.2^59^ within a Singularity environment. RNA-seq reads from leaf tissues were aligned to the genome using STAR, followed by transcript assembly across samples with StringTie and subsequent refinement and validation of transcript models using PASA. Two sets of *ab initio* gene predictions were generated using BRAKER3 v3.0.8^60^ incorporating the RNA-seq evidence and using Helixer v0.3.4^61^ employing the land_plant model. All evidence was integrated using EvidenceModeler (EVM) v2.0.0^62^ to produce a high-confidence consensus gene set. The final annotation included 146,216 protein-coding genes across all four haploid chromosome sets of the tetraploid genome with 660 genes from non-chromosomal scaffolds (Fig. 7; Table 1).

Repeat annotation was conducted using EDTA v2.2.2^63^ for *de novo* identification and construction of a genome-specific TE library. Candidate elements were further classified using DeepTE^64^, a deep-learning-based tool that improves TE classification accuracy, particularly for non-LTR elements and unclassified TEs. The curated repeat library was used with RepeatMasker v 4.1.5^65^ to comprehensively annotate repetitive elements across the genome. In total, 10,854,626 repeats were identified, covering 79.8% of the genome, with the LTR-Gypsy retrotransposon being the most abundant, comprising 21.2% of the assembly (Fig. 7; Table 4).

**Table 4:** Summary of repetitive sequences in the creeping bentgrass genome.

Class	Order	Family	Copy number	Length (Mb)	% of Genome
Class I (Retrotransposon)	LTR	Copia	470,300	232.5	4.28
		Gypsy	1,677,404	1150.3	21.19
		Unknown	153,688	76.5	1.41
	non-LTR	DIRS	7,141	2.4	0.04
		PLE	37,186	11.1	0.2
		SINE	41,753	3.5	0.06
		LINE	97,945	60.9	1.13
		Unknown	83,173	35.3	0.65
Class II (DNA transposon)	TIR	P-element	3,219	2.8	0.05
		Tc1/Mariner	678,832	153.8	2.83
		hAT	784,619	194.5	3.58
		Harbinger	802,495	286.1	5.26
		CACTA	1,527,195	486.1	8.96
		Mutator	1,963,951	829.5	15.27
	non-TIR	Helitron	98,750	26.1	0.48
		Mite	211,630	22.9	0.42
		Unknown	944,280	312.1	5.75
Low Complexity			33,330	1.9	0.03
Simple Repeat			304,742	14.8	0.27
Unknown			932,993	429.0	7.9
Total			10,854,626	4331.8	79.79

### Functional annotation

Functional annotation of protein-coding genes was performed using the Trinotate pipeline v4.0.0^66^. To assign putative functions, DIAMOND BLASTP^67^ searches were conducted against the UniProtKB/Swiss-Prot protein database with an e-value cutoff of 1e-5. The resulting hits were used to infer functional homology. Protein domains were identified using HMMER v3.4^68^ against the Pfam-A database. Gene Ontology (GO) terms and KEGG pathway assignments were derived from matches to UniProtKB/Swiss-Prot and Pfam entries. Overall, 126,238 genes (86.3%) received at least one functional annotation based on homology, domain structure, or pathway association (Table 5).

**Table 5:** Summary of functional annotation for predicted protein-coding genes.

Annotation category	Number of genes	% of predicted genes
Total predicted protein-coding genes	146,216	100.0%
Genes with BLASTP hits	98,969	67.7%
Genes assigned to KEGG Pathways	81,671	55.9%
Genes with Pfam domain Hits	121,651	83.2%
Genes with GO terms from BLASTP	95,433	65.3%
Genes with GO term from Pfam	75,142	51.4%
Genes annotated with any source (overall)	126,238	86.3%

## Data Records

All raw sequencing data have been deposited in the NCBI Sequence Read Archive (SRA). Illumina paired-end RNA-seq data (SRX30375246-SRX30375263) are available under SRA accession SRP617872^69^. PacBio Iso-Seq HiFi reads (SRX30389376–SRX30389377), PacBio HiFi genomic reads (SRX30389372–SRX30389373, SRX30389378–SRX30389382), Oxford Nanopore long reads (SRX30389374, SRX30389383–SRX30389385), and Omni-C Illumina data (SRX30389375) are deposited under SRA accession SRP618122^70^. The assembled genome is available through NCBI Datasets under accession GCA_052724435.1 (haploid, haplotype 1)^71^ and GCA_052724335.1 (haploid, haplotype 2)^72^. Genome assemblies, predicted gene models, and transposable element annotations are publicly available via AgData Commons and can be accessed at 10.15482/USDA.ADC/30081199.v2^73^.

## Technical Validation

Genome completeness was evaluated using BUSCO (v5.8.2) against the poales_odb12 database (6,282 single-copy orthologs). The assembled genome achieved a BUSCO completeness score of 98.8%, while the annotated gene set showed 99.5% completeness. To further assess assembly quality, BUSCO analysis was also performed with each subgenome, yielding completeness scores of 97.4–97.8% for the subgenomes and 98.2–98.3% for subgenome gene annotations. These results indicate that both the genome assembly and its gene annotation are highly complete and of high quality.

## Data Availability

All data processing commands and pipelines were executed in accordance with the instructions and guidelines provided by the respective bioinformatic software. No custom scripts or code were used in this study.
